# Five willow varieties cultivated across diverse field environments reveal stem density variation associated with high tension wood abundance

**DOI:** 10.3389/fpls.2015.00948

**Published:** 2015-10-31

**Authors:** Nicolas Berthod, Nicholas J. B. Brereton, Frédéric E. Pitre, Michel Labrecque

**Affiliations:** ^1^Institut de Recherche en Biologie Végétale, University of Montréal, MontrealQC, Canada; ^2^Institut de Recherche en Biologie Végétale, Montreal Botanical Garden and University of Montréal, MontrealQC, Canada

**Keywords:** biofuels, willow (*Salix* sp.), tension wood, density, wood anatomy

## Abstract

Sustainable and inexpensive production of biomass is necessary to make biofuel production feasible, but represents a challenge. Five short rotation coppice willow cultivars, selected for high biomass yield, were cultivated on sites at four diverse regions of Quebec in contrasting environments. Wood composition and anatomical traits were characterized. Tree height and stem diameter were measured to evaluate growth performance of the cultivars according to the diverse pedoclimatic conditions. Each cultivar showed very specific responses to its environment. While no significant variation in lignin content was observed between sites, there was variation between cultivars. Surprisingly, the pattern of substantial genotype variability in stem density was maintained across all sites. However, wood anatomy did differ between sites in a cultivar (producing high and low density wood), suggesting a probable response to an abiotic stress. Furthermore, twice as many cellulose-rich G-fibers, comprising over 50% of secondary xylem, were also found in the high density wood, a finding with potential to bring higher value to the lignocellulosic bioethanol industry.

## Introduction

Renewable energy, such as lignocellulosic biofuel derived from sustainably sourced biomass, could play an important role in offsetting the deleterious global impacts of fossil fuel use. Biomass combustion has long been used to produce heat and electricity, and already accounts for more than 10% of global energy consumption (IPCC, 2011). Plant species with the potential for particularly high biomass yields in temperate climates include short rotation coppice (SRC) willow (Labrecque and Teodorescu, 2003). While being high biomass yielding, SRC willow also has a number of advantageous properties in terms of sustainability and environmental impact, such as low fertilizer requirements, high stress tolerance (surviving on marginal or even polluted land) as well as positive effects on biodiversity (Labrecque et al., 1993; Karp and Shield, 2008).

Wood is the majority of harvested biomass and is composed of xylem rich in fiber cells with thick secondary cell walls. These secondary cell walls are composed of a complex cross-linked polymer matrix including: lignin, cellulose and hemicelluloses; together endowing the plant with properties such as: strength and protection against pathogens. Easy access to the sugar monomers within the cell wall matrix to be used for fermentation into ethanol is necessary to make biofuel production feasible, but represents a challenge (Taherzadeh and Karimi, 2008; Zhu et al., 2010; Limayem and Ricke, 2012). Lignin is not the targeted polymer in either the pulp and paper or biofuel industries, so reducing its content without compromising tree integrity is often thought to be important to increase process efficiency (Simmons et al., 2010). However, in addition to reducing lignin content, improvement to the accessibility of cellulose would also benefit such industries by reducing processing energy requirements. Mechanical stimuli, such as wind, have been shown to induce modification of wood anatomy in willow, which creates a unique type of xylem tissue tension wood (TW) aligned with the direction of the mechanical force. Recently, this TW has been found to be rich in accessible cellulose, highly beneficial biofuel production in allowing reduced process energy to be used in releasing cell wall glucose (Bowling and Vaughn, 2008; Brereton et al., 2011, 2012). The majority of the accessible cellulose within TW is though to lie within a cell wall layer unique to TW, the gelatinous-layer (or G-layer), which is internal to the secondary cell wall of secondary xylem fiber cells.

In addition, soil properties can also affect plant chemistry and morphology. Limitation or excess of nutrients such as potassium, nitrogen, or magnesium is shown to alter plant phyisiology (Shaul, 2002; White and Karley, 2010). Although variant soil magnesium (Ache et al., 2010) and potassium (Fromm, 2010) soil concentrations are likely to alter wood formation in trees such as willow little research has been directly reported other than the effect of nitrogen (Pitre et al., 2010) which has been shown to promote TW formation.

Willows show substantial genotypic variability in regard to biomass production (Labrecque and Teodorescu, 2005; Volk et al., 2006). However, when the fate of biomass is biofuel, the ability to release cell wall sugar is also of great importance as well as biomass yield alone (Brereton et al., 2011, 2012). Moreover, the impact of environmental factors such as stress (drought, salinity, pollution) or stimuli (wind, radiation) on growth and developmental strategy may also vary by genotype (Marmiroli et al., 2011; Weih et al., 2011; Serapiglia et al., 2013a).

Wood density is an important factor to consider in the context of biomass production for bioenergy as high density biomass can translate into high heating value (Goel and Behl, 1996). Less is known about the importance of density relating to biofuel potential. Wood composition and tree architecture both influence density (Serapiglia et al., 2013a; Brereton et al., 2015). A number of developmental factors could individually or collectively influence wood architecture: cell division, cell expansion as well as tissue variation (Zanne et al., 2010). Density in each genotype could therefore vary in response to different environmental factors in different ways. However, to date only a few studies have examined wood density in relation to wood composition and architecture in fast growing woody species (*Populus, Salix*) from contrasting environments (Mansfield and Weineisen, 2007; Brereton et al., 2012).

The aim of this study was to investigate the wood composition, anatomy, and density variability of five genotypes of 2-year-old willow stems on four different sites in the province of Québec (Canada).

## Materials and Methods

### Experimental Sites

**FIGURE 1:**
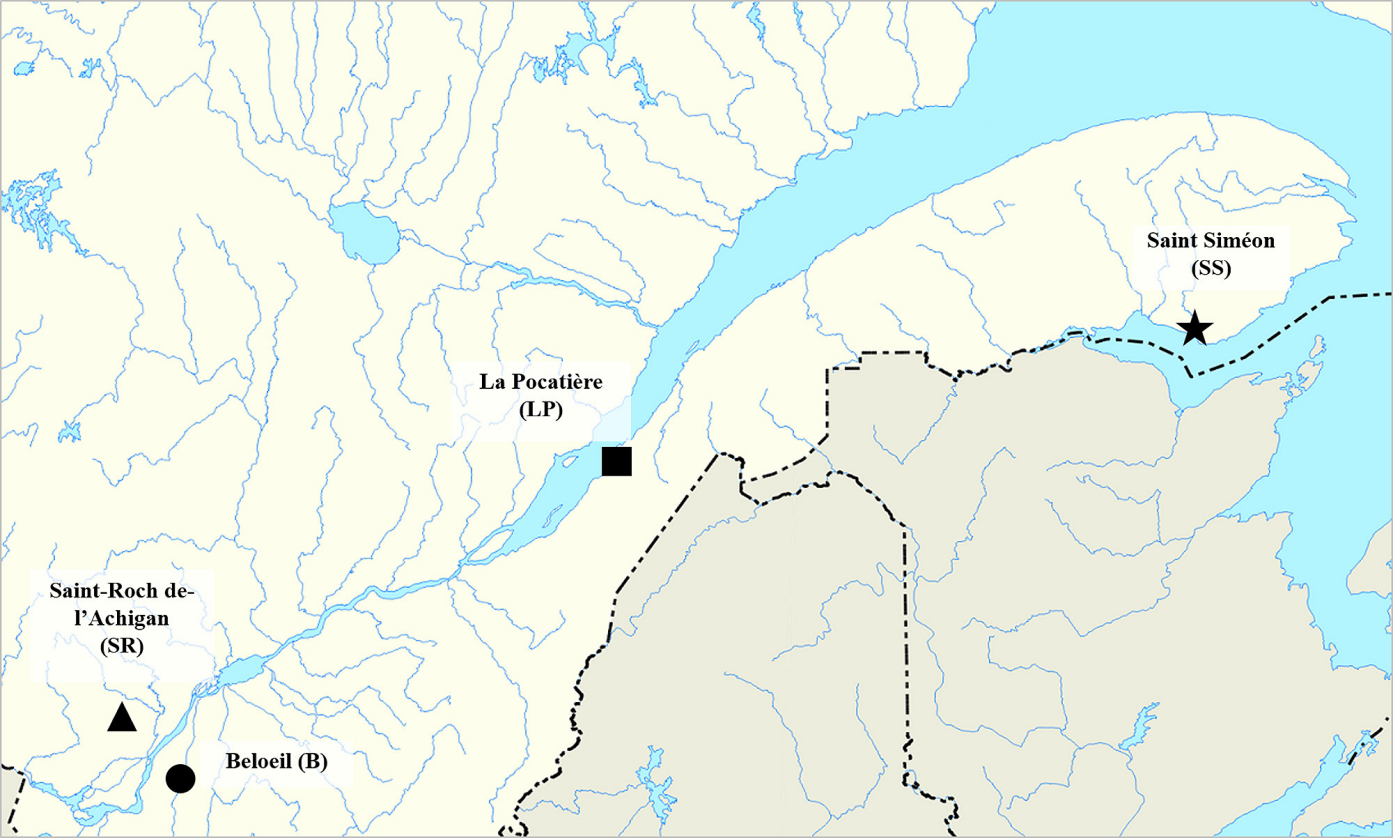
**Site localization in Québec province, Canada: Saint-Roch-de-l′Achigan (SR), Beloeil (B), Saint-Siméon (S-S), and La Pocatière (LP)**.

The willow plantations were established in the spring of 2011 by the *Réseau des Plantes Bio-industrielles du Québec* (RPBQ), a network to develop bio-industrial crops in the Province of Québec (Canada). The experiments were carried out at four sites representing a range of environmental conditions along the Saint Lawrence River near the communities of Beloeil (B) 45°35′32.8 N – 73°14′46.7 W, Saint-Roch-de-l′Achigan (SR) 45°48′56.5 N – 73°39′08.8 W, La Pocatière (LP) 47°21′05.1 N – 70°01′35.6 W. and Saint-Siméon (SS) 48°05′11.2 N – 65°35′11.1 W (**Figure 1**).

Five high biomass yielding (Labrecque and Teodorescu, 2005; Zalesny et al., 2011) willow genotypes were established at each of the sites: *Salix viminalis* 5027, *S. dasyclados* SV1, *S. miyabeana* SX61, *S. miyabeana* SX64, *S. miyabeana* SX67. Each of the sites comprised four randomized blocks, each block consisting of 20 trees per genotype, totalling 400 trees per site. Soil from each site was sampled in 2011 by RPBQ with measured soil characteristic provided in **Table 1.** Climate data was collected from Environment Canada weather stations in 2013 (**Table 1**). Fertilizer high in nitrogen was applied to all sites to buffer site variation (100 kg N per hectare).

**Table 1:** Comparison of site characteristics (single soil sample analyzed in 2011 and climate data collected for 2013 from Environment Canada) at each of the four sites (Saint-Roch-de-l′Achigan, Beloeil, Saint-Siméon (S-S) and LP).

		Units	Saint Roch de l′Achigan	Beloeil	La Pocatière (LP)	Saint-Siméon
	**Texture**		**Loamy Clay**	**Loamy Clay**	**Clay**	**Sandy Loam**
Soil^∗^	Organic matter	wt%	4,15	4,2	5,61	2,07
	pH		7,66	7,3	6,09	5,24
	Available P	kg/ha^-1^	99	31	101	50
	Available K	kg/ha^-1^	160	729	475	155
	Available Ca	kg/ha^-1^	11547	7570	5889	1662
	Available Mg	kg/ha^-1^	354	2490	1138	72
	Al	kg/ha^-1^	1781	2576	2180	2453
Climate	Annual average monthly max. t°C	°C	20,2	20,9	18,6	16,7
	Annual average monthly min. t°C	°C	-11,1	-8,7	-9,2	-8,6
	Annual average t°C	°C	4,9	6,0	3,8	3,7
	Growing degree days		1194,2	1204,7	794,6	699,0
	Total precipitations	mm	1194,9	885,6	770,6	934,3
	Annual average daily max. wind	km/h	62,7	≤31	74,9	65,2


*^∗^depth 0–20 cm.*

### Tree Morphology and Density

Six trees per cultivar, in the second year of their harvest cycle, were sampled randomly within a single block. Stem number, the tallest stem and largest stem base diameter was measured for each tree.

Two classical methods for determining stem density were compared using water displacement to assess stem volume; basic density and oven dry specific gravity (Williamson and Wiemann, 2010) using biomass from two of the sites, B and SS (which preliminary tests suggested varied in density). Density, which varied substantially between sites, reflected the same pattern regardless of method type. Basic density was 15–23% lower than oven dry specific gravity in absolute amount in all cases. Oven dry specific gravity alone was used for the remainder of the samples.

### Wood Composition

Wood samples were air dried before being ground through a 40-mesh (<525 μm). Extractives were determined following the standard procedures ASTM D1107-16 and ASTM D1110-84. Ten grams of wood were extracted first with toluene/ethanol and then with water over a period of 7 h using Soxhlet apparatus.

Lignin content was determined following standard Klason procedures ASTM D1106-96 for acid insoluble lignin and NREL LAP-004 for acid soluble lignin. Briefly, 300 mg of extractives free sample was hydrolysed in 72% sulfuric acid for 2 h at 30°C before dilution with 84 mL water and autoclaving at 120°C for 1 h. Samples were then filtered; acid insoluble lignin being determined by weight whereas acid soluble lignin was determined via spectrometry. Phenolic content was extracted by sonication of 500 mg of sample in methanol, and separation by centrifuged for 15 min at 4800 rpm repeated three times. Phenolics were then quantified using the modified Prussian blue colorimetric method (Price and Butler, 1977; Graham, 1992), using UV spectrophotometer absorbance at 700 nm compared to a gallic acid standard.

### Microscopy

Stem samples of *Salix miyabeana* SX64 were collected in the summer of 2014 at LP (the site with lowest wood density values) and at Saint-Roch de-l′Achigan (highest density values). Samples were fixed in FAA (Formaldehyde – Acetic Acid – Ethanol) and sectioned at 40 μm using a sledge microtome. Sections were then stained with 1% aqueous Safranin O (staining lignified cell wall) and 1% Chlorazol Black E in methoxyethanol (staining cellulose). Image analysis was conducted using QCAD drawing software and Image J image analysis software using triplicate biological samples for each site.

Transverse section g-fibers coverage was measured using four different methods. Method A (manual) involved manual drawing of stained g-fiber area using QCAD. Method B (pixel distribution) quantified the number of pixels binned by grayscale (an intensity scale of 0 to 254; zero being black, 254 being white) in a bin chosen based on observed pixel distribution (0 to 100), of transverse wood sections from 3 trees per site (one from each of three blocks, chosen randomly from the four) using Image J. Method C (blind distribution) quantified the number of pixels in the same manner, binned by grayscale (0 to 254) in a bin chosen blindly as half the scale intensity (0 to 127). Method D (black and white) was used to count black pixels, stained with chlorazol black, of monochrome (Black/White) transverse wood images, again using Image J.

### Statistics Analysis

Analyses of variance (ANOVA) followed by Tukey’s honestly significant difference (HSD; α < 0.05) were performed to determine statistical differences of diameter, height, density, wood composition, and anatomical data. All analyses were made using JMP (SAS Institute, Inc.).

## Results

### Tree Morphology and Composition

**FIGURE 2:**
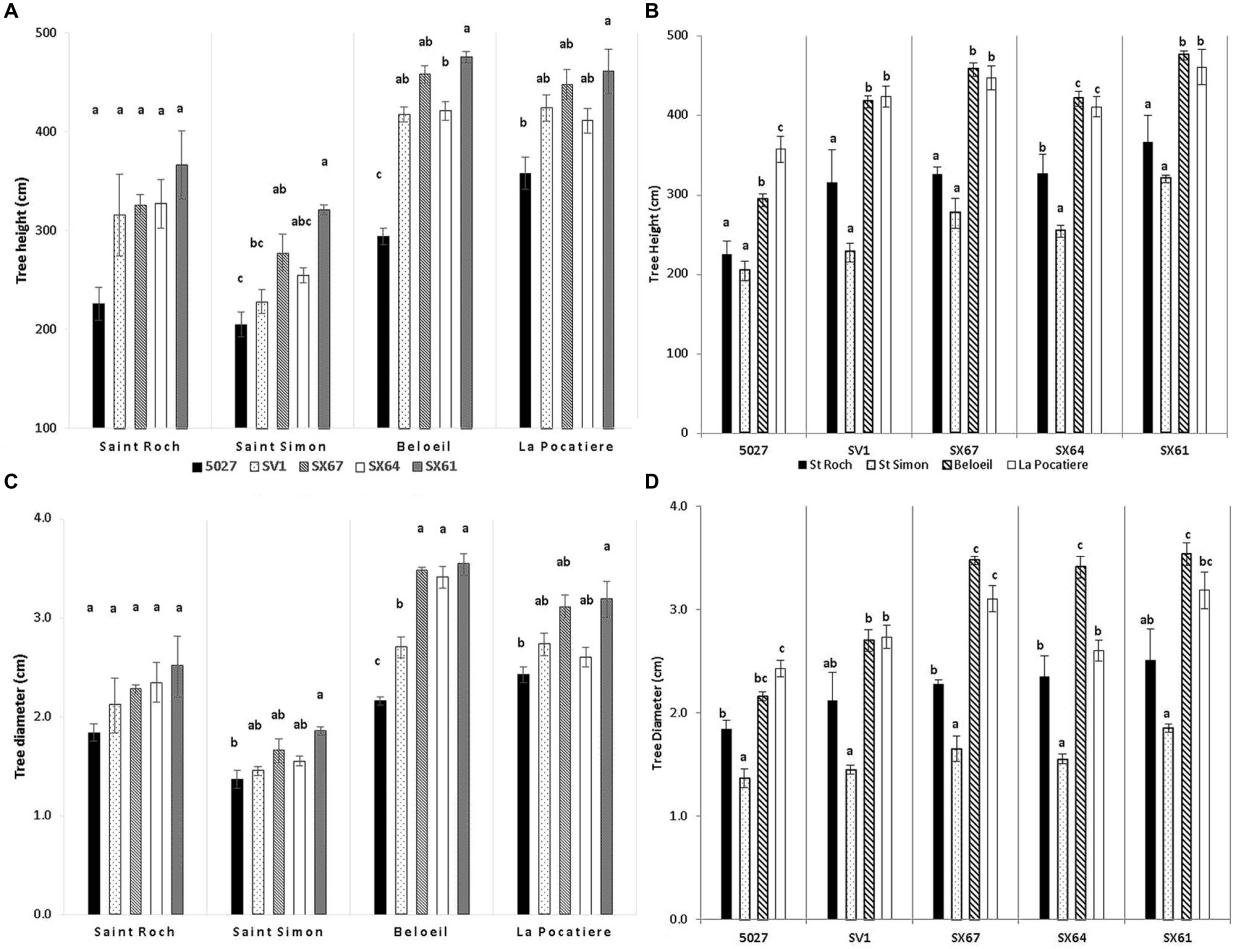
**Tree height (**A** – by site; **C** – by genotype) and stem diameter (**B** – by site; **D** – by genotypes) from five willow cultivars in their second year of a harvest cycle (*Salix viminalis* 5027, *S. dasyclados* SV1, *S. miyabeana* SX61, *S. miyabeana* SX64, *S. miyabeana* SX67) sampled at four field trials in Québec: Saint-Roch-de-l′Achigan, Beloeil, S-S, and LP.** Error bars represent standard error, *n* = 4 blocks (6 trees per block). Tukey′s Honestly Significant Difference (HSD) pairwise *post hoc* test (α = 0.05) are represented by letters a–c.

The environmental climate of each of the four sites varied (**Table 1**) in regard to temperature, precipitation, wind and growing degree days (GDD correspond to days with temperatures higher than 5°C) (McMaster and Wilhelm, 1997). B and SR had the largest number of GDD and warmer annual temperatures than LP and SS. At LP and Beloeil (B), trees were significantly (*p* < 0.05) taller (420 and 413 cm, respectively) than those at Saint-Roch-de-l′Achigan (SR; 312 cm) and SS (257 cm) when all trees were averaged regardless of genotype (**Figure 2A**). Stem diameter at B and LP was also significantly (*p* < 0.05) larger (31 and 28 mm, respectively) than at SR (22 mm) and SS (16 mm; **Figure 2B**). There was a general trend between genotypes across the four sites: genotype SX61 had larger stem diameters (27.7 mm SE 3.7) and heights (405.7 cm SE 37.2) than the other cultivars. However, comparing genotypes within site, this pattern did not always persist. For example, at SR, no significant variation was observed between genotypes for diameter or for height, except in 5027 (**Figure 2**).

**FIGURE 3:**
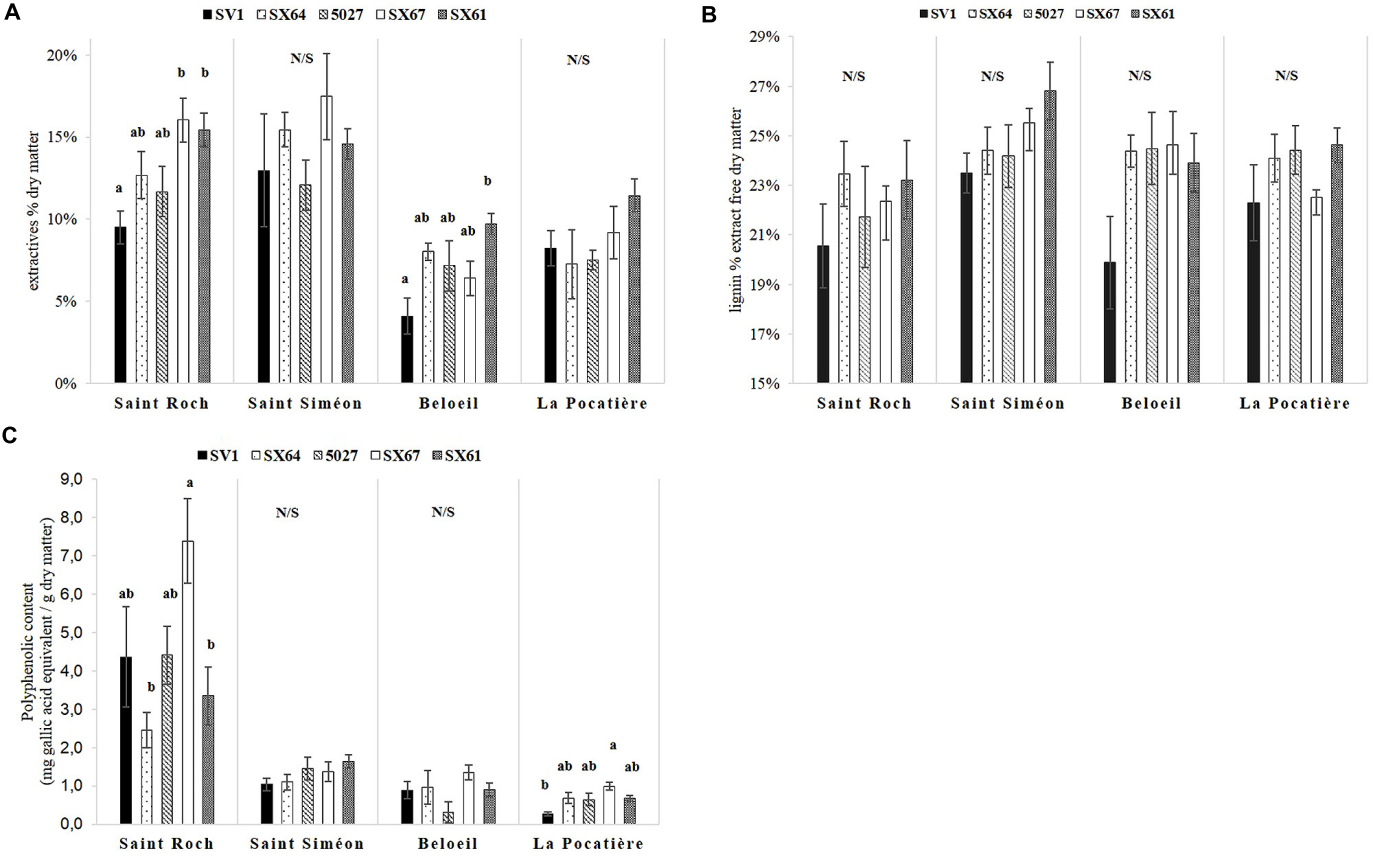
**Five willow cultivars in their second year of a harvest cycle (*Salix viminalis* 5027, *S. dasyclados* SV1, *S. miyabeana* SX61*, S. miyabeana* SX64, *S. miyabeana* SX67) sampled at four field trials in Québec: Saint-Roch-de-l′Achigan, Beloeil, S-S and LP.** **(A)** Variation in extractives (toluene-ethanol/water extraction); **(B)** lignin content expressed as a percentage of dry matter and **(C)** total polyphenolics content (methanol-extractable). Error bars represent standard error, *n* = 4 blocks (four trees per block). Tukey’s HSD pairwise *post hoc* test (α = 0.05) are represented by letters a–b.

Wood extractives content varied significantly (*p* < 0.05, ANOVA *F*-test) between genotypes and sites. Extractives were higher in all trees cultivated at SS (14.5% *SE* 0.5) and SR (13.1% *SE* 0.1) (**Figure 3A**), almost double that of B and LP, which had 7.1 and 8.7% extractives, respectively. Within extractable compounds, substantially more polyphenols were produced from a single site (SR) than any other, and by a single genotype (SX67), with 4.28 mg (total for the five cultivars at SR) and 7.39 mg (SX67 average at SR) g^-1^ gallic acid equivalent, respectively, (**Figure 3C**). Significant differences in total lignin content were not observed between or within the sites (**Figure 3B**).

### Density

**FIGURE 4:**
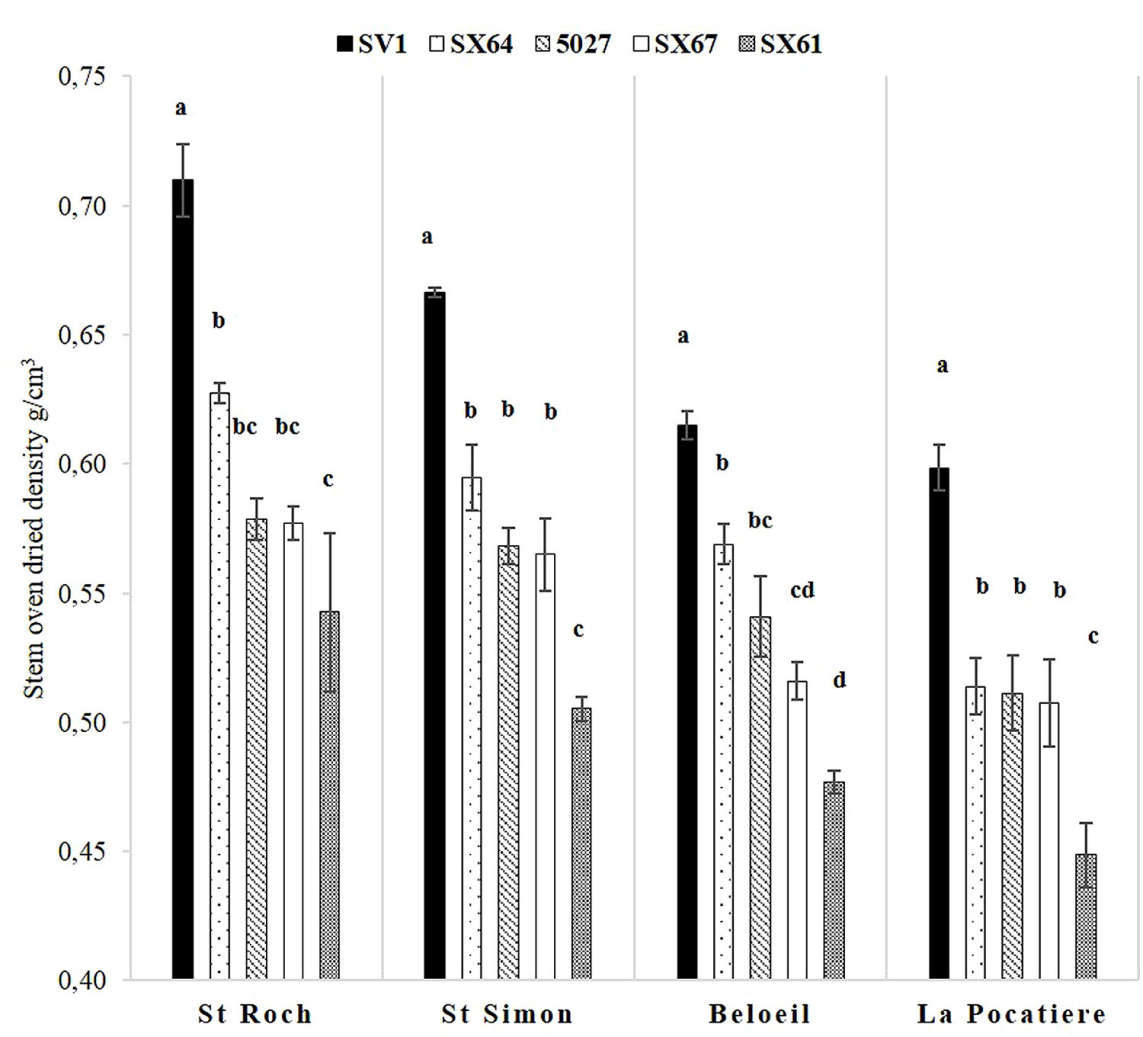
**Stem density was assessed using oven dried specific gravity (grams per cubic centimeters) from five willow cultivars in their second year of a harvest cycle (*Salix viminalis* 5027, *S. dasyclados* SV1, *S. miyabeana* SX61, *S. miyabeana* SX64, *S. miyabeana* SX67) sampled at four field trials in Québec: Saint-Roch-de-l′Achigan, Beloeil, S-S and LP.** Error bars represent standard error, *n* = 4 blocks (four trees per block). Tukey’s HSD pairwise *post hoc* test (α = 0.05) are represented by letters a–d.

When all the genotypes in a site were averaged, trees cultivated at SR and SS had significantly (*p* < 0.05, ANOVA *F*-test) denser wood stems, with means of 0.61 g/cm^3^ and 0.58 g/cm^3^, respectively, compared to B and LP, with means of 0.54 g/cm^3^ and 0.52 g/cm^3^ (**Figure 4**). The same genotype rank order (1- SV1; 2- SX64; 3/4- 5027/SX67; 5- SX61) was consistent at every site but there was substantial variation in density between sites. Considering only those sites with the highest and lowest densities, SR ranged from 0.71 g/cm^3^ (SV1) to 0.54 g/cm^3^ (SX61), whereas at LP they ranged from 0.59 g/cm^3^ (SV1) to 0.44 g/cm^3^ (SX61) revealing *Salix*, grown under different conditions and of different genotype, has at least the capacity to increase by 60% in density. Stem height and density was significantly (*p* < 0.05) negatively correlated between trees only at SS (if genotype effect is discounted) with an r value of – 0.61.

### Wood Anatomy

**FIGURE 5:**
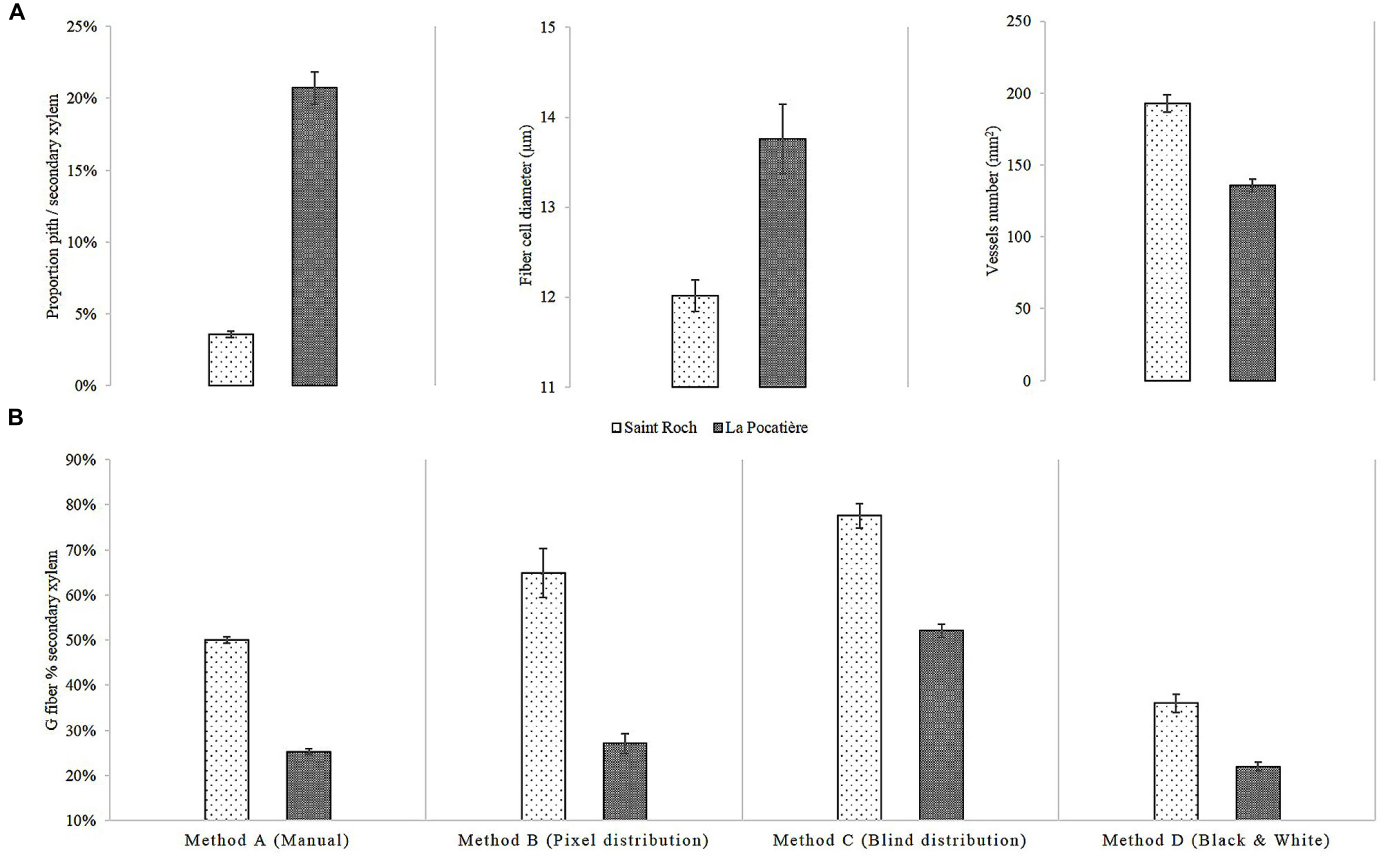
**SX64 cultivar (*S. miyabeana*) in its third year of a harvest cycle sampled at two field trials in Québec: Saint-Roch-de-l′Achigan and LP.** **(A)** Measured Anatomical traits: (i) pith proportion, (ii) fiber cell diameter, and (iii) vessels number per 1 mm2; **(B)** Variation of G fibers determined by different image analytical methods: Method A (Manual) involved manual drawing around stained g-fiber, Method B (Pixel distribution) quantified pixels in a bin from 0 to 100, Method C (Blind distribution) quantified pixels in a bin from 0 to 127 and Method D (Black and White) count black pixels of monochrome. Error bars represent standard error (*n* = 3 trees, one per randomized block). All comparisons between Saint Roch and La Pocatiere presented in this figure are significantly different (*t*-test, *p* < 0.05).

Wood anatomy was characterized from three trees of a single genotype, SX64, from each of two sites: LP (lowest density site) and SR (highest density site). The genotype was chosen as it varied the most in terms of density between these two sites (**Figure 4**). The pith to secondary xylem ratio of the SX64 stems at LP were over five times larger (20.7% SE 1.12, of the transverse area) than those of SR (3.6% SE 0.23) (**Figure 5A**). There were more vessels per surface area unit (1 mm^2^) at SR (193, compared to 136 at LP), whereas fibers had a larger average diameter at LP, 13.8 μm as opposed to 12 μm at SR.

**FIGURE 6:**
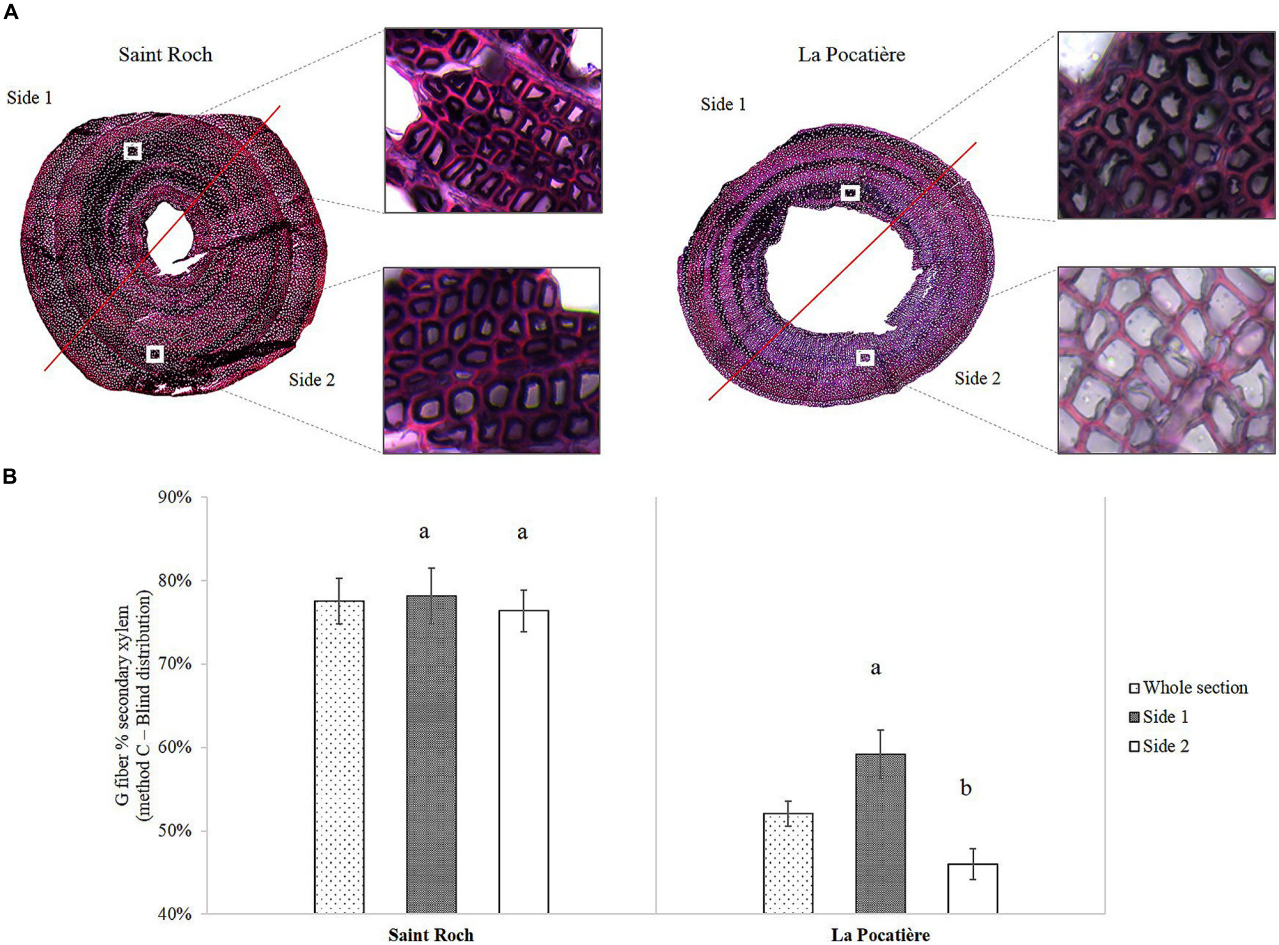
**(A)** 40 μm transverse section of a stem from SX64 cultivated at Saint-Roch de l′Achigan (left – most dense) and at LP (right – least dense). Stained in 1% Chlorazol Black E in methoxyethanol and 1% aqueous Safranin O. **(B)** Polarization of G fibers determined by Method C (Blind distribution) from SX64 cultivar (*S. miyabeana*) in its third year of a harvest cycle sampled at two field trials: Saint-Roch-de-l′Achigan and LP. Error bars represent standard error (*n* = 3 trees, one per randomized block). *t*-test pairwise *post hoc* test (α = 0.05) are represented by letters a–b.

We determined gelatinous fiber (g-fiber) abundance (a characteristic of TW) through specific staining of the gelatinous-layer (g-layer) and image analysis of the biologically replicated samples (*n* = 3). As this type of site comparison of proportional (net stem) g-fiber quantification is relatively novel for mature field-grown trees (to our knowledge, most having been pot studies to date), four different methods were assessed to ensure confidence in quantification. Based on the results of these four methods, wood grown in SR had significantly and substantially more g-fibers than LP (**Figure 5B**). Based on method A, SR had twice as many observed g-fibers, with 50% of the tissue being g-fiber containing compared to 25% at LP. Using method C, transverse polarization (i.e., Tissue patterning) of the g-fibers were determined by dividing the wood stem section into two parts (**Figure 6A**). Significant and substantial variation in tissue patterning was observed between sites, with TW always being produced somewhere across the stem at SR but with no clear polarization (no clear “upper” or “lower” side of the stem from a transverse perspective) (**Figure 6B**). At the LP site (which uniformly produced less dense wood as well as substantially less TW), clear polarization was observed with no TW produced during some points of the growing season, no significant TW polarization was observed in the high density wood from the SR site.

## Discussion

### Site Influence on Growth and Wood Composition

Tree development alters in response to the surrounding environment. Multiple abiotic and biotic factors can affect this response in woody plants, including nutrient availability, soil, climate, competitors, predators, and available space (Kozlowski, 1997). In our trials, B and LP showed similar morphological growth, with trees on these sites becoming taller and greater in diameter than those at SS and SR. The number of GDD has been correlated (positively) to biomass production in Finland by Tahvanainen and Rytkönen (1999). However, here, if we consider height and diameter to be predictors of biomass yield, such a relationship is not obvious as SS and SR represent the different extremes of the spectrum for GDD. Located near the Saint Lawrence River, LP is reported to be a comparatively windy site. As wind has been shown to sometimes affect tree morphology, inducing development of shorter and stronger trees in a process called thigmomorphogenesis (De Langre, 2008), LP could be expected to display such morphology, surprisingly this was not the case. Since fast growing willows have high water requirements (Dawson, 2007), the lower amount of precipitation recorded on the LP site may reflect inadequate water supply. However, precipitation during this growing season should have been sufficient for willow’s needs (>430 mm) according to data reported by Lindroth and Cienciala (1996) whilst such climatic differences between sites are clear, in terms of variation in genotype development, the single most influential environmental factor could not be separated here.

B and SR present similar climatic conditions but distinct soil properties. SR soil contained comparatively less potassium and magnesium than B and LP, two elements important in plant physiology (Shaul, 2002; White and Karley, 2010). SR is located on former agricultural land where a large amount of calcium carbonate was added to the soil to reduce aluminum toxicity associated with acidity (Rengel, 1992). Calcium was therefore found to be much more abundant on this site than on the others. While this nutrient is beneficial for plants, playing a role in intracellular signal and wood formation (Lautner and Fromm, 2010), it is potentially cytotoxic in excess, affecting roots in soil and reducing growth rates (White and Broadley, 2003). Comparing sites LP and B, the former had a phosphorus level (essential in many compound molecules such as nucleotides, phospholipids and sugar phosphates (Rennenberg and Herschbach, 2013) three times that of B. Both potassium and phosphorus can have an impact on tree growth; Hytönen and Kaunisto (1999) reported that combined phosphorus and potassium fertilization can increase willow biomass yields by 64%. With smaller diameter and shorter trees comparatively, SS was also found to have acidic soil, with less organic content, potassium, phosphorus, and magnesium, which suggests a deleterious impact of the nutrients at these concentrations on growth.

Surprisingly, lignin values were not significantly different between the four sites (**Figure 3B**), suggesting the cell wall response to environment here may not be driven by variation in lignin content. Swan and Kellogg (1986) also found no significant variation in lignin content of poplar collected from three different locations in British Columbia, Canada. Serapiglia et al. (2013b) also found only two out of 17 willow cultivars had significant differences in lignin content between two contrasting environments in New York State (USA). This is in stark contrast to clear variation in lignin content between genotypes observed in other willow studies (Serapiglia et al., 2009; Stolarski et al., 2011; Ray et al., 2012; Zamora et al., 2014). It is interesting to note such large changes in wood development and architecture without substantial (net) effects to an important cell wall element such as lignin.

Trees grown at SR had more extractable polyphenols than those grown at other sites. Polyphenol biosynthesis can be induced in trees by stress, such as biotic stress related to herbivore attack (Gershenzon, 1984; Tahvanainen et al., 1985; Constabel et al., 2000). Water and nutrient availability in soil have also been shown to affect polyphenol concentrations in such trees; Price et al. (1989) and Osier and Lindroth (2001) observed phenolic glycoside variation in willow was dependant on soil nutrient availability. No extensive insect herbivory was reported during the growing season at SR, it is therefore possible, given the high calcium concentration measured at the site, that the extraordinary abundance in polyphenols could be driven by soil properties or nutrient availability. Variation in extractives was also observed between sites in our trials. Increases in extractives at SR, compared to B and LP, might be explained by the abundance of phenolics. Trees on SS also contained more extractives than those at B and LP, however, phenolic content was not greatly increased (as in SR), implying variation of non-phenolic extractables such as: sugars, proteins, phospholipids, or other secondary metabolites.

### Genotypic Variation among Wood Traits

Over the 2-year cycle from 2012 to 2013, SX61 grew over 30% taller and greater in diameter than 5027, which suggests that the former genotype should be preferentially selected if diameter and height were used as a proxy for biomass yield. Genotype selection is an important first step for developing high-performing crops suitable for biomass or biofuels. Willow cultivars have previously been shown to exhibit broad differences in biomass productivity (Labrecque and Teodorescu, 2005; Tharakan et al., 2005). In the field, Serapiglia et al. (2013a) identified a genotype x environment interaction on two contrasting sites. However, height and stem diameter may not be measures that represent biomass most accurately, considering the extreme variation in density.

Extractable polyphenols are a potential source of high value molecules (tannins, salicylic acid) and, once extracted from wood and purified, could generate additional revenues for biomass and biofuel industries (Boudet, 2012). One specific cultivar, SX67, produced larger amounts of polyphenols than the other genotypes (**Figure 3C**), indicating that genotypes could be selected to increase yields of such high value molecules (however, site effect was had the greatest influence polyphenolic yields).

### Density Variability of Wood Properties

Wood density is a common measure in the forest industry because it represents a useful parameter for calculations related to biomass transport and feedstock processing. In ecology, it is frequently used as a predictor of carbon allocation and mechanical strength (Hacke et al., 2001; Chave et al., 2009). Large differences in wood density were identified among the four study sites. Wood from LP and B was, in general, less dense than that from SR and SS.

Basic density was negatively correlated with the growth traits of stem height in trees grown at SS (if the large effect of genotype was discounted), a relationship repeatedly seen in other trials (Muller-Landeau, 2004; Pliura et al., 2007; Chave et al., 2009). The differences observed in density, both between sites and genotypes (**Figure 4**); suggest that, in this case, height and diameter would not be accurate indicators for predicting biomass yield in willow. In fact, considering the negative correlation of stem height and diameter with the extreme differences in density, they may actually directly contradict real yields. Destructive harvesting across all genotypes and sites is needed to definitively establish the usefulness of these proxies in predicting biomass yield and these density data suggest such research is necessary.

A similar genotype pattern for density was observed across all sites: SV1 (highest density), SX64, 5027, SX67, and SX61. Cultivar variation in wood density has been previously reported in willow (Sennerby-Forsse, 1985; Mosseler et al., 1988) and in poplar (Klasnja et al., 2003) and variability between sites was observed in previous studies (Pliura et al., 2007), suggesting a genotype-specific response in relation to the environment. The strong and consistent behavior of genotypes observed here for this trait led us to explore anatomy in the hope of revealing the origin of such patterning. One of the major anatomical factors known to greatly affect density in willow is reaction wood formation (Brereton et al., 2015).

### Relationship between Stress and Wood Anatomy

The developmental response of trees to mechanical stress by altering wood morphology, called thigmomorphogenesis, has been extensively studied (Telewski and Jaffe, 1981; Jaffe and Forbes, 1993). Telewski (1990) observed increases in wood density aligned with increased ethylene produced in pines, and associated such increases in ethylene to the trees response to mechanical disturbance (Telewski and Jaffe, 1986). One explanation for the large variation in wood density here could be the difference in pith size found between SR and LP. As well as this, another substantial anatomical difference was observed (**Figure 6A**). Wind is thought to be one of the principle inducers of mechanical stress in the environment, often mimicked in greenhouse experiments by bending or tipping trees, and results in the production of a specific type of wood, termed reaction wood, often characterized by a cellulose rich tissue layer called TW (Jourez et al., 2001; De Langre, 2008; Brereton et al., 2011, 2012).

The site at LP, defined as a comparatively windy site, was found to have a large and polarized proportion of TW (presumably localized to the “upper” part of the stem), suggesting the influence of “directional” mechanical force (**Figure 6A**). Although potentially subjected to higher mechanical stress (via increased wind speeds), surprisingly, LP had half the TW of SR. TW was observed in extraordinary amounts in wood from SR, with over 50% of wood on average in all the samples from this high density genotype grown in SR containing the cellulose rich g-fibers. Recent evidence suggests that the cellulose present in gelatinous fibers (g-fibers) of TW is easily accessible to digesting enzymes and may be of high value to the lignocellulosic biofuel industry (Brereton et al., 2012). TW production has almost exclusively been reported as a response to a mechanical or gravity stimulus. The comparatively low wind speed and lack of clear polarization at SR (**Figure 6B**) suggests induction of reaction wood was not wind induced as the classical model would presume. This type of adaptation within wood anatomy, driven by environmental stress, might point to a new source of TW induction: non-mechanical stress.

An increase in the number of vessels and decrease in fiber cells was also found at SR (**Figure 5A**), a pattern described by Escalante-Perez et al. (2009) in poplar exposed to salinity stress; their change in wood structure was explained by the effect of K+ on cell expansion. Less expansion can result in smaller fiber cell diameters, imparting greater density in the xylem as a whole. Wood exposed to high salinity environments has been shown to have altered vessel morphology, resulting in their reduced diameters (Junghans et al., 2006) and to result in reinforcement of secondary cell walls, increasing their thickness (Hacke et al., 2001). The interdependence of vessel morphology (for hydraulic architecture) and fiber morphology (for mechanical structure) has recently been suggested as potentially linking traits such as water stress and TW production (Brereton et al., 2015). Altered salt concentrations have also directly been shown to interact with TW formation by Janz et al. (2012) who found that poplars subjected to salt stress formed a novel type of wood, ‘pressure wood’, where genes know to be involved in TW formation were down-regulated. The authors also noted up-regulated elements of the phenylpropanoid pathway, in line with the high polyphenol content found at SR and so providing a clue as to the potential origin of such high TW production.

## Conclusion

The environment of willow cultivation had large effects on both tree morphology and internal wood structure. These effects were complicated by genotype-specific responses in traits such as tree height, stem diameters, polyphenolic content as well as substantial differences in density between the four sites investigated. Surprisingly, given the complex genotype-specific variation, wood density seemed a uniquely consistent trait in that the ranking of genotypes remained constant across all the sites, suggesting consistent genetic regulation.

Although net lignin content of wood did not vary to any considerable extent, TW was observed in extraordinary amounts in high density wood of a specific genotype. While the direct benefit to cell wall polysaccharide accessibility needs to be explored, such drastic changes to cellulose construction throughout the entirety of the wood, without any observable detriment to the growth of these fully mature crops, presents an exciting opportunity to exploit natural developmental responses to improve crop selection for biofuel production.

## Conflict of Interest Statement

The authors declare that the research was conducted in the absence of any commercial or financial relationships that could be construed as a potential conflict of interest.
